# Utility of tolvaptan sodium phosphate for refractory fluid retention in post-transplant sinusoidal obstruction syndrome

**DOI:** 10.1007/s12185-025-04022-z

**Published:** 2025-06-06

**Authors:** Koshi Akahane, Shin Kasai, Minori Tamai, Yukihiro Sugita, Hiroko Oshiro, Kumiko Goi, Takeshi Inukai

**Affiliations:** Department of Pediatrics, School of Medicine, University of Yamanashi, 1110 Shimokato, Chuo, Yamanashi 409-3898 Japan

**Keywords:** Hematopoietic stem cell transplantation, Sinusoidal obstruction syndrome, Fluid retention, Ascites, Tolvaptan sodium phosphate

## Abstract

Sinusoidal obstruction syndrome (SOS) is a life-threatening complication of hematopoietic stem cell transplantation (HSCT), particularly in patients with a high HokUS-10 score after starting treatment. Tolvaptan sodium phosphate (TSP) is a novel intravenous aquaretic agent used to treat refractory fluid retention in congestive heart failure (CHF). Here, we report the successful treatment of severe post-HSCT SOS with refractory fluid retention and CHF using TSP plus defibrotide. A 22-year-old man with relapsed acute lymphoblastic leukemia underwent unrelated peripheral blood stem cell transplantation and developed SOS on day 13. Despite defibrotide therapy and standard management, fluid retention rapidly progressed, resulting in an 18.3% increase in body weight on day 21 and a high HokUS-10 score (10/13 points). TSP (16 mg) administered to treat the CHF immediately induced adequate urine output. Continued TSP treatment (8 mg/day) resulted in sustained diuresis and a return to baseline body weight on day 33. The only significant adverse event observed during the 5 weeks of TSP treatment was transient hypernatremia (148 mEq/L). Defibrotide was discontinued on day 72 because the HokUS-10 score had decreased to 1 point. Our experience suggests the utility of TSP in controlling refractory fluid retention due to post-HSCT SOS.

## Introduction

Sinusoidal obstruction syndrome (SOS) is a serious and potentially life-threatening complication of hematopoietic stem cell transplantation (HSCT). The diagnosis of post-HSCT SOS is made based on clinical criteria consisting of the presence of painful hepatomegaly, jaundice, ascites, and weight gain [1]. Severe SOS typically results in multi-organ dysfunction and is associated with higher mortality rates [2]. Although the introduction of defibrotide therapy has dramatically reduced mortality in severe SOS cases [3, 4], careful management of fluid balance remains critical. In particular, since massive ascites and pleural effusion due to fluid retention occasionally cause severe respiratory distress requiring ventilatory support and/or drainage, early diagnosis and prompt treatment are critical. Diuretics such as furosemide and spironolactone are commonly used to control fluid retention but are sometimes ineffective in severe SOS cases [2, 5, 6].

Tolvaptan is a vasopressin V_2_ receptor antagonist that acts on renal collecting tubules to increase free water excretion (aquaresis) [7]. Oral tolvaptan is widely used in the treatment of refractory fluid retention associated with heart failure or liver cirrhosis. Notably, the utility of oral tolvaptan for a case with severe fluid retention due to post-HSCT SOS was previously reported, but the long-term outcome was inconclusive because the patient reportedly died on day 55 after HSCT due to rapid progression of lymphoma [8]. Meanwhile, tolvaptan sodium phosphate (TSP) is a recently developed intravenous prodrug of tolvaptan with improved water solubility. It has been approved in Japan for the treatment of congestive heart failure (CHF) refractory to conventional diuretics [9, 10], based on a phase III OPTION-HF trial that confirmed the non-inferiority of intravenous TSP (16 mg once daily) to oral tolvaptan (15 mg once daily) [10].

In this report, we present a case of post-HSCT SOS complicated by refractory fluid retention and CHF, which were rapidly and safely controlled by TSP. To the best of our knowledge, this is the first case report describing the utility of TSP in controlling severe fluid retention associated with post-HSCT SOS.

## Case report

A 22-year-old Japanese male was admitted with relapsed B-cell precursor acute lymphoblastic leukemia (BCP-ALL), which had initially presented when he was 13 years old. Although refractory to conventional reinduction chemotherapy, hematological complete remission (CR) was achieved after two cycles of inotuzumab ozogamicin (InO) monotherapy. He subsequently achieved minimal residual disease-negative CR after CD19-targeted chimeric antigen receptor T-cell therapy. During these treatments, he experienced frequent premature ventricular contractions (PVCs) and transient ventricular tachycardia, so oral potassium supplementation was started to prevent hypokalemia. Three months after the last dose of InO, the patient underwent unrelated peripheral blood stem cell transplantation (uPBSCT) from a 6/8 HLA-matched female donor. Following myeloablative conditioning with total body irradiation (TBI, 12 Gy) and melphalan (140 mg/m^2^), donor peripheral blood stem cells (3.3 × 10^6^/kg of CD34-positive cells) were infused with graft-versus-host disease (GVHD) prophylaxis using tacrolimus and short-course methotrexate. Ursodeoxycholic acid (UDCA, 400 mg/day), heparin (10 units/kg/hour), and lipo-prostaglandin E1 (lipo-PGE1, 30 μg/day) were administered for SOS prophylaxis.

**Fig. 1:**
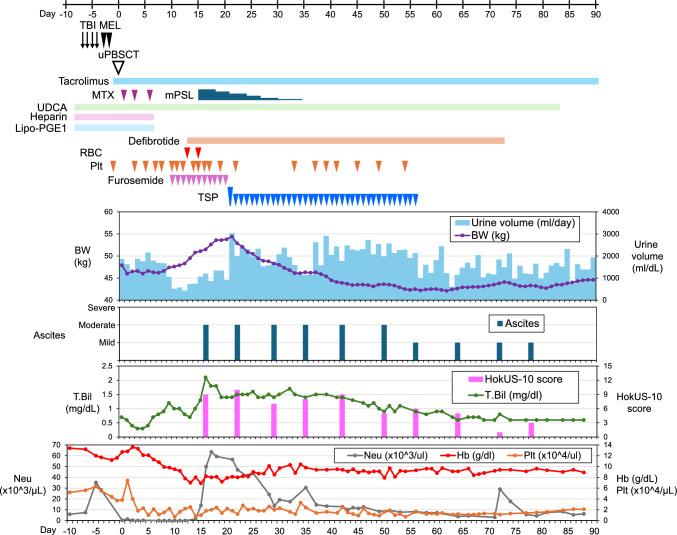
Clinical course after the conditioning regimen. The degree of ascites was evaluated by abdominal echography. *TBI* total body irradiation, *MEL* melphalan, *MTX* methotrexate, *mPSL* methylprednisolone, *UDCA* ursodeoxycholic acid, *RBC* red blood cell transfusion, *Plt* platelet transfusion, *BW* body weight, *T.Bil* total bilirubin, *Neu* neutrophil count

**Fig. 2:**
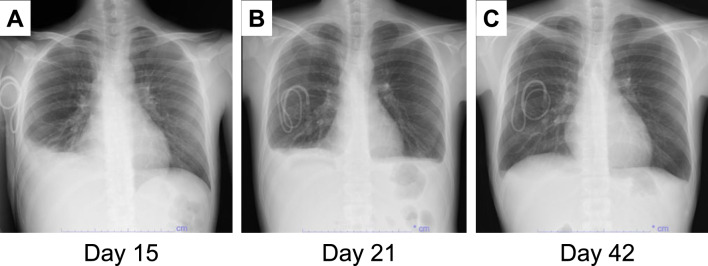
Changes in chest radiographic findings. Chest radiographs on day 15 (**A**), day 21 (**B**) and day 42 (**C**) after uPBSCT are shown

On day 4 after uPBSCT (Fig. 1), the patient developed transfusion-refractory thrombocytopenia. On day 7, he developed an extensive subcutaneous hematoma on the right upper arm, so both heparin and lipo-PGE1 were discontinued. Daily administration of intravenous furosemide was started on day 10 due to decreased urine output, but his body weight increased from 46.0 to 49.5 kg (7.6% increase) on day 13, accompanied by painful hepatomegaly and ascites. Thus, a clinical diagnosis of SOS was made according to the modified Seattle criteria, and defibrotide therapy was initiated. Concomitant with neutrophil engraftment (neutrophil counts over 500/µl) on day 15, he developed engraftment syndrome with fever, tachypnea, and skin rash, which resolved after administration of methylprednisolone (1 mg/kg/day). A chest radiograph on day 15 revealed cardiomegaly and pulmonary congestion with right pleural effusion, consistent with a diagnosis of CHF (Fig. 2A). On day 16, his total bilirubin level increased to 2.1 mg/dL, meeting the European Group for Blood and Marrow Transplantation 2023 criteria for clinical SOS [1]. We evaluated his HokUS-10 score, which measures 10 parameters of abdominal ultrasound findings and detects SOS with 100% sensitivity and 95.8% specificity when a score is 5 points or higher [11]. His HokUS-10 score was 9 points on day 16. Despite fluid restriction and daily administration of intravenous furosemide, his ascites and edema progressed with bilateral pleural effusions on day 21 (Fig. 2B). His body weight further increased to 51.1 kg (11.1% increase) on day 15 and to 54.4 kg (18.3% increase) on day 21, accompanied by severe abdominal distension and orthopnea. In addition, PVCs frequently appeared from day 20. Neither intensified furosemide nor spironolactone was available due to the risk of hypokalemia and the interaction with tacrolimus, respectively. His renal function remained normal (serum creatinine level: 0.46 mg/dL), and he had no clinical and laboratory signs of pulmonary or central nervous system dysfunction. Under these circumstances, to control CHF and severe fluid retention, we administered 16 mg of TSP on day 21. Notably, his urine output increased to 1,900 mL within 6 h after the first dose of TSP and eventually reached 3,038 mL/day, with a concomitant reduction in body weight by 1.5 kg in 1 day. Although mild hypernatremia (148 mEq/L) was observed transiently on day 21, his serum sodium concentration was normalized by day 22. TSP administration (8 mg/day) was continued from day 22, resulting in sustained urine output (1130–2910 mL/day) and return of body weight to baseline by day 33. A chest radiograph on day 42 showed the resolution of pleural effusions (Fig. 2C). His HokUS-10 score was 10 points [moderate ascites and mean portal vein (PV) velocity < 10 cm/s] on day 22 and decreased to 7 points (moderate ascites and mean PV velocity ≧10 cm/s) on day 29 (Fig. 1). Finally, a significant reduction in ascites was observed on day 56, and TSP was discontinued (Fig. 1). No significant adverse events were observed during TSP therapy, except for transient hypernatremia after the first dose of TSP, and no PVCs occurred after day 41. In addition, the plasma tacrolimus concentrations were stable (5.9–11.5 ng/mL) without significant dose adjustments, and no acute GVHD was observed. Even after cessation of TSP, his HokUS-10 score decreased to 1 point on day 72, and defibrotide therapy was discontinued. Regarding engraftment of uPBSCT, complete donor chimerism in peripheral blood cells was confirmed on day 21 by fluorescence in situ hybridization (FISH) analysis of the sex chromosomes. His neutrophil and platelet counts remained relatively low, but platelet transfusions were no longer required after day 54. Finally, complete donor chimerism was confirmed by FISH analysis of sex chromosomes in the bone marrow aspirate on day 79. Twelve months after uPBSCT, the patient remained in CR, with no signs of hepato-renal dysfunction associated with SOS.

## Discussion

Severe fluid retention is a life-threatening feature of SOS due to the induction of ascites and pleural effusion. As standard treatments for fluid retention, fluid restriction, sodium management, and administration of diuretics such as furosemide and spironolactone are recommended [2, 6]. However, the management of severe cases refractory to standard treatment remains clinically challenging. In the present report, we describe the successful control of severe fluid retention and CHF due to post-HSCT SOS using TSP in combination with defibrotide. Considering several risk factors in the present case, including HSCT from an unrelated donor, large donor/recipient HLA disparity, high-dose TBI-based conditioning, and prior exposure to InO [2], we administered UDCA, heparin, and lipo-PGE1 to prevent SOS. However, the patient developed SOS, and his fluid retention progressed rapidly despite the early initiation of defibrotide therapy and standard supportive care, including fluid restriction and daily intravenous furosemide. A recent retrospective study of post-HSCT SOS showed that SOS-related mortality was approximately 60% in the cases with a higher HokUS-10 score (≥ 8 points) after initiation of SOS treatment [12]. In our case, the highest HokUS-10 score was 10 points on day 22, despite the initiation of defibrotide therapy on day 13 with standard supportive care, suggesting a poor prognosis. Of clinical importance, although it took approximately 8 weeks to resolve SOS completely, TSP administration resulted in sustained urine output and rapid improvement of fluid retention, which could causally contribute to overcoming severe SOS.

To the best of our knowledge, this is the first case report on the utility of TSP for severe post-HSCT SOS. However, a previous single case report suggested the efficacy of oral tolvaptan for post-HSCT SOS with cardiopulmonary failure [8]. In this previous case, oral administration of tolvaptan for 2 days (3.75 mg and 7.5 mg) effectively controlled ascites and edema. As a minor adverse event, although hypernatremia gradually developed from 135 to 159 mEq/L within 3 days after the last administration of oral tolvaptan, it was reportedly controlled with dextrose infusion. Despite the recovery of the reverse PV flow, as indicated on echography, the patient reportedly died of lymphoma progression on day 55. Thus, although the long-term outcome was inconclusive, this report suggests the possible efficacy of aquaretic agents for severe fluid retention associated with post-HSCT SOS.

Compared to oral tolvaptan, intravenous TSP has several advantages, including rapid onset of action, flexibility in dose adjustment, and utility in cases of impaired oral intake [9, 10, 13, 14]. Of note, the time to maximum plasma concentration of active tolvaptan in intravenous administration of TSP is reportedly approximately 1.5 h, while that in oral administration of tolvaptan itself is 4 h [9]. As a result, in CHF patients with refractory fluid retention, TSP reportedly exerts a diuretic effect within 1 h after administration and reaches a peak effect within 1 or 2 h [15]. Consistently, in our case, although his urine output was only 920 mL/day 1 day before TSP administration, it reached 1900 mL within 6 h after the first dose of TSP, resulting in rapid clinical improvement of fluid retention.

In the present case, TSP appeared to effectively relieve severe fluid retention primarily through its diuretic effect. Although the rapid diuresis induced by TSP improved hemodynamic parameters, TSP might not directly improve the sinusoidal endothelial damage and inflammatory cascades associated with SOS. Further pathophysiological studies are necessary to clarify the pharmacological effects of TSP in SOS.

Electrolyte abnormalities are a known adverse effect of TSP due to hemoconcentration caused by excessive aquaresis. In the OPTION-HF study, the incidence of hypernatremia in the TSP-treated cases (2.7%) was almost similar to that in the oral tolvaptan-treated cases (2.1%) [10]. In contrast, the incidence of hyperkalemia and dehydration was higher in the TSP-treated cases (6.0% and 10.1%, respectively) than in the oral tolvaptan-treated cases (2.1% and 4.1%, respectively) [10]. In this context, it is noteworthy that nearly half of the cases that developed hyperkalemia after TSP treatment had underlying renal dysfunction, suggesting a requirement for careful monitoring, particularly in cases with renal dysfunction. In our case, although mild hypernatremia developed after an initial dose of TSP, it resolved the next day without any therapeutic intervention. Ultimately, no other adverse effects were observed during 5 weeks of TSP administration.

In the post-HSCT setting, potential interactions between TSP and other drugs are an important clinical issue. TSP is rapidly converted to tolvaptan, which is a substrate of P-glycoprotein (P-gp) and cytochrome P450 3 A4 (CYP3 A4) [16, 17]. Although no significant dose adjustments of tacrolimus were necessary during TSP therapy in the present case, further accumulation of clinical data is needed to evaluate potential interactions with other drugs such as tacrolimus, a substrate of P-gp and CYP3 A4 [18, 19], and cyclosporine, a potent inhibitor of P-gp and CYP3 A4 [20].

In conclusion, our case report demonstrates the potential utility of TSP in controlling severe and refractory fluid retention due to post-HSCT SOS, although further clinical studies are required to verify its efficacy and safety.

## Data Availability

The data are not publicly available due to privacy restrictions.
